# Beyond State of the Art: Digital psychiatry is coming of age!

**DOI:** 10.1192/j.eurpsy.2023.29

**Published:** 2023-07-19

**Authors:** H. Riper

**Affiliations:** ^1^Clinical Psychology, VU University; ^2^Psychiatry, A-UMC, Amsterdam, Netherlands

## Abstract

**Abstract:**

Digital interventions for common mental disorders are coming of age. The uptake of evidence-based self-guided and guided (a-synchronic in time) cognitive behavioural digital interventions for depression and anxiety in routine care is however low. Blended treatment formats appear an attractive alternative for routine care settings as these combine face-to-face sessions with digital ones in integrated treatment protocols. Yet little is known about the clinical and cost-effectiveness of blended CBT (bCBT). In this presentation I will touch upon the current state of the art of digital interventions for depression and anxiety disorders, including blended formats in routine care and I will go beyond that state by addressing new developments. I will illustrate these new developments by virtue of several studies (RCT’s) we conducted on the clinical and cost-effectiveness of blended CBT interventions (and their implementation) in routine care settings. A specific focus will be put on blending digital phenotyping in CBT treatment which includes mobile ecological momentary assessment tools and AI algorithms.

**Conclusion:**

While digital mental health for depression and anxiety in routine care was boosted through the Covid-19 pandemic, the question remains whether the current knowledge base will leveraging digital mental health research and services to the next level. Implications for future research on blended treatments and clinical applications in routine care will be discussed.

**Disclosure of Interest:**

None Declared

